# Lime in Food

**Published:** 1875-05

**Authors:** 


### LIME IN FOOD.

The fact that superfine flour contains only one-fifth as much phosphate of lime as is found in the entire grain, affords an important suggestion as to the selection of our material for bread. Without a due proportion of this salt —phosphate of lime—in the food, the bones and teeth are imperfectly formed. It is an *indispensable* constituent of these structures. It is as needful for the child as for the mature man, and even more so.

The system of the nursing babe, in whom the teeth and bones are in their formative process, must be furnished with this salt of lime or "rickets," and other indications of imperfect nutrition are likely to occur.

The babe, dependent on the mother for its nutriment, suffers from every error in her diet. It is therefore of the greatest importance that the mother should, in the selection of her food, never forget the wants of her child. If she lives on food containing phosphates, the milk will not lack this important constituent. Phosphate of lime is found in nearly all our food, unless it has been removed by some process of art; as when in the manufacture of superfine flour, that portion of the wheat which contains the most of this salt is separated and thrown away. It is said by a late writer, that the use of artificial preparations of the lime-salts will not compensate for their deficiency in food. These salts must be in their natural combinations with vegetable substances or they are not assimilated. If taken in medicinal preparations they are rejected, and pass out of the system with the other excreta. It seems that Nature, in building the house we live in, prefers materials in the shape that she has prepared them, rather than those which come from the laboratory of the chemist and the pharmacist.

The importance of a liberal proportion of such articles as unbolted flour, oatmeal, etc., in our daily food, cannot be overestimated. And especially mothers, nursing their babes (as all mothers ought to do , should partake freely of this kind of diet, for reasons mentioned above.

